# The effects of endurance exercise in hypoxia on acid-base balance and potassium kinetics: a randomized crossover design in male endurance athletes

**DOI:** 10.1186/s40798-018-0160-1

**Published:** 2018-10-13

**Authors:** Daichi Sumi, Chihiro Kojima, Nobukazu Kasai, Kazushige Goto

**Affiliations:** 1 Graduate School of Sports and Health Science, Ritsumeikan University, Kusatsu, Shiga Japan; 2 Faculty of Sports and Health Science, Ritsumeikan University, 1-1-1, Nojihigashi, Kusatsu, Shiga 525-8577 Japan

**Keywords:** Hypoxia, Endurance exercise, Acid-base balance, Potassium

## Abstract

**Background:**

Exercise-induced disturbance of acid-base balance and accumulation of extracellular potassium (K^+^) are suggested to elicit fatigue. Exercise under hypoxic conditions may augment exercise-induced alterations of these two factors compared with exercise under normoxia. In the present study, we investigated acid-base balance and potassium kinetics in response to exercise under moderate hypoxic conditions in endurance athletes.

**Methods:**

Nine trained middle-to-long distance athletes [maximal oxygen uptake (VO_2max_) 57.2 ± 1.0 mL/kg/min] completed two different trials on different days, consisting of exercise in moderate hypoxia [fraction of inspired oxygen (F_i_O_2_) = 14.5%, H trial] and exercise in normoxia (F_i_O_2_ = 20.9%, N trial). They performed interval endurance exercise (8 × 4 min pedaling at 80% of VO_2max_ alternated with 2-min intervals of active rest at 40% of VO_2max_) under hypoxic or normoxic conditions. Venous blood samples were obtained to determine blood lactate, pH, bicarbonate ion, and K^+^ concentrations before exercise, during exercise, and after exercise.

**Results:**

The blood lactate concentrations increased significantly with exercise in both trials. Exercise-induced blood lactate elevations were significantly greater in the N trial than in the H trial at all time points (*P* = 0.012). Bicarbonate ion concentrations (*P* = 0.001) and blood pH (*P* = 0.019) during exercise and post-exercise periods were significantly lower in the N trial than in the H trial. A significantly greater exercise-induced elevation in blood K^+^ concentration was produced in the N trial than in the H trial during exercise and immediately after exercise (*P* = 0.03).

**Conclusions:**

High-intensity interval exercise on a cycle ergometer under moderate hypoxic conditions did not elicit a decrease in blood pH or elevation in K^+^ levels compared with an equivalent level of exercise under normoxic conditions.

## Key points

- High-intensity interval exercise on a cycle ergometer under moderate hypoxic conditions did not elicit the decline of blood pH compared with an equivalent level of exercise under normoxic conditions.
- Exercise-induced elevations in blood lactate concentrations were significantly greater in normoxia than in hypoxia.
- The exercise under moderate hypoxic conditions did not elicit the K^+^ elevation compared with the exercise under normoxic conditions.

## Background

The use of exercise training in normobaric hypoxia (hypoxic training) has been widely accepted as a potent tool for improving the endurance capacity of athletes, and a large amount of experimental evidence supports the efficacy of this training method [1–4]. Exercise under hypoxic conditions triggers lower systemic oxygen uptake [5] and arterial oxygen saturation [6], whereas carbohydrate utilization [7, 8] and exercise-induced blood lactate levels [9, 10] are augmented. These specific responses during exercise under hypoxic conditions may trigger improvement of exercise performance and promote peripheral adaptation in the muscles. However, the mechanism underlying these adaptations is not fully understood. As previously discussed, energy supply via the glycolytic system is enhanced under hypoxic conditions [9, 10], which results in a further decrease in blood pH. Exercise-induced acidosis is considered as a limiting factor for sustained power output during exercise [11–13]. Once protons (hydrogen ions) are produced in the working muscles and subsequently released into the bloodstream, the protons are buffered mainly by the bicarbonate (HCO_3_^−^) buffering system [14, 15]. Therefore, the exercise-induced kinetics of blood HCO_3_^−^ reflect the homeostasis of acid-base balance in the blood during intensive exercise.

In addition to acid-base balance, extracellular potassium (K^+^) accumulation is proposed to attenuate exercise tolerance, because it markedly decreases muscle excitability [16–18]. Exercise under hypoxic conditions requires increased anaerobic energy supplies [19], with a concomitant increase in the blood lactate concentration [9, 10]. These metabolic responses increase the opening of the muscular ATP-sensitive K^+^ (K_ATP_) channels [20–22]. Therefore, exercise under hypoxic conditions, with greater intramuscular and blood acidification, promotes the exercise-induced increase of blood K^+^ concentration compared with exercise under normoxic conditions. In contrast, higher blood lactate and K^+^ concentrations during exercise may stimulate an increased capacity to maintain acid-base balance and K^+^ homeostasis during exercise [23–25]. Mohr et al. (2006) compared the effects of two different intense training regimens on skeletal muscle ion transport proteins and exercise performance. Sprint endurance training (30-s runs), with greater disturbance of muscle ion homeostasis (higher blood lactate and K^+^ concentrations) during training sessions, caused greater adaptations of Na^+^/H^+^ exchanger isoform 1 and the Na^+^/K^+^-ATPase α2 isoform and further improved exercise performance, compared with sprint training (6-s sprints). Thus, the determination of acid-base balance and K^+^ kinetics during exercise and post-exercise would greatly improve the understanding of the mechanism underlying enhanced exercise capacity after hypoxic training periods [1–4].

Therefore, the purpose of the present study was to evaluate acid-base balance and K^+^ kinetics in endurance athletes during and after exercise under moderate normobaric hypoxic conditions. We hypothesized that endurance exercise under moderate hypoxic conditions would facilitate exercise-induced metabolic acidosis and elevate K^+^ levels compared with the same relative exercise intensity under normoxic conditions.

## Methods

### Subjects

Nine endurance athletes participated in the present study. The age, height, and body mass [mean ± standard error (SE)] of the subjects were 20.1 ± 0.4 years, 173.6 ± 2.7 cm, and 65.6 ± 2.0 kg, respectively. All athletes were born and currently living at sea level, and they were performing specific training in middle-to-long distance running 5 days per week (approximately 70 km per week). They were informed of the experimental procedures and possible risks involved in this study, and written informed consent was subsequently obtained. The study was approved by the Ethics Committee for Human Experiments at Ritsumeikan University (BKC-IRB-2016-037), and it was conducted in accordance with the Declaration of Helsinki [26].

### Experimental design

The subjects visited the laboratory four times during the experimental period. During the first and second visits, two maximal oxygen uptake (VO_2max_) tests were completed using a graded power test on a cycle ergometer (Aerobike 75XLIII; Konami Corporation, Tokyo, Japan) under normoxic conditions [inspired oxygen fraction (F_i_O_2_) = 20.9%] or normobaric hypoxic conditions (F_i_O_2_ = 14.5%, a simulated altitude of 3000 m).

**Fig. 1:**
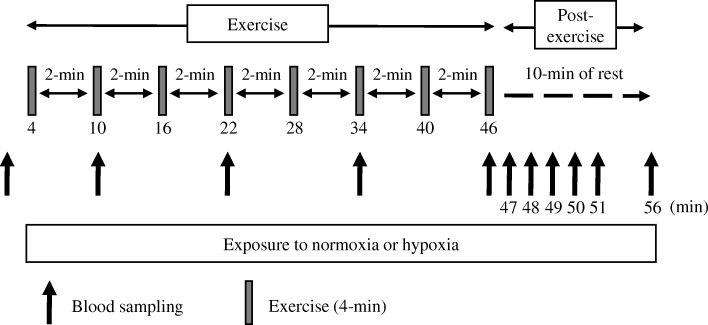
Overview of the study design

On the third and fourth occasions, subjects carried out experimental trials under either hypoxic conditions (F_i_O_2_ = 14.5%, H trial) or normoxic conditions (F_i_O_2_ = 20.9%, N trial). As shown in Fig. 1, all subjects completed high-intensity interval exercise on a cycle ergometer (Aerobike 75XLIII; Konami Corporation, Tokyo, Japan) at the same relative exercise intensity relative to VO_2max_ evaluated under the hypoxic or normoxic conditions. After completing the exercise, subjects rested for 10 min in the respective conditions. Venous blood samples were obtained before, during (sets 2, 4, 6, and 8), and after exercise (1, 2, 3, 4, 5, and 10 min after completing exercise). Expired gases, heart rate (HR), and rate of perceived exertion (RPE) were measured during exercise. The two trials were spaced at least 1 week apart. Furthermore, the two trials were performed at the same time of the day, and the order of the trials was randomized.

### Exercise protocols

In the H and N trials, all exercise sessions were conducted on an ergometer (Aerobike 75XLIII; Konami Corporation, Tokyo, Japan). After a 5-min warm-up at 50% of VO_2max_, the subjects started interval endurance exercise (8 × 4 min pedaling at 80% of VO_2max_ with 2 min of active rest at 40% of VO_2max_ between sets) under hypoxic or normoxic conditions. In the present study, pedaling workload was determined relative to VO_2max_ evaluated under normoxic or hypoxic conditions. The hypoxic chamber used in the present study was the whole-room type, and the hypoxic condition was established by diluting ambient air with nitrogen [10, 27]. The pedaling frequency was kept constant at 80 r/min. To avoid psychological influence, F_i_O_2_ and workload were not displayed to the subjects.

### Measurements

#### Maximal oxygen uptake

The VO_2max_ test was conducted twice under normoxic and hypoxic conditions. The test began at 100 W, and the load was increased progressively by 30-W increments every 2 min until exhaustion (80 r/min). During the test, expired gases were collected and analyzed using an automatic gas analyzer (AE300S, Minato Medical Science Co., Ltd., Tokyo, Japan). The respiratory data were averaged every 30 s. HR was measured continuously during the test using a wireless HR monitor (Accurex Plus; Polar Electro Oy, Kempele, Finland). The order of the two repeated bouts of VO_2max_ tests under normoxic and hypoxic conditions was randomized. These tests were performed at least 3 days apart.

#### Blood variables

Following an overnight fast, the subjects visited the laboratory at 7:30 a.m. and rested before the first blood collection. A 22-gauge polyethylene catheter was inserted into an antecubital vein after a 20-min rest, and a baseline blood sample was obtained. A series of blood samples were collected at sets 2, 4, 6, and 8 during interval exercise (blood was drawn at 3–3.5 min during each set). During the post-exercise period, further blood samples were collected at 1, 2, 3, 4, 5, and 10 min after exercise completion. All blood samples used for determining blood gas and electrolyte levels were collected using 2.5-mL syringes containing heparin. A 5-mL syringe was utilized to obtain serum samples.

Blood glucose, lactate, HCO_3_^−^, potassium (K+), and sodium (Na+) concentrations; pH; and oxygen (pO_2_) and carbon dioxide partial pressure (pCO_2_) were measured using an automatic blood-gas analyzer (OPTI CCA TS, Sysmex Co., Hyogo, Japan). These analyses were completed within 15 min after blood collection, and the samples were kept on ice until analysis. Blood glucose and lactate concentrations were measured using a glucose analyzer (FreeStyle, Nipro Co., Osaka, Japan) and a lactate analyzer (Lactate Pro, Arkray Co., Kyoto, Japan) immediately after blood collection.

#### Cardiorespiratory variables and RPE

Oxygen uptake (VO_2_), carbon dioxide output (VCO_2_), respiratory exchange ratio (RER), and expired minute ventilation (VE) were determined during exercise using the breath-by-breath method. During the 8 × 4-min interval exercise session, the average respiratory variable values were calculated during the final minute of each 4-min set. HR and percutaneous oxygen saturation (SpO_2_) were recorded at the same time points as those of the respiratory gas samplings. The SpO_2_ was recorded every 1 s using a finger pulse oximeter (Pulsox-Ma300, Teijin, Tokyo, Japan) placed on the tip of the right forefinger. The subjects indicated their rating of respiratory strain (RPE-R) and leg strain (RPE-L) at the end of each set of exercises using a 10-point scale measuring perceived exertion [28].

### Statistical analyses

Data are expressed as means ± SD. Two-way analysis of variance (ANOVA) with repeated measures was used to assess the presence of interactions (trial × time) and main effects (trial, time). When ANOVA revealed a significant interaction or main effect, the Tukey–Kramer test was performed as a post hoc analysis to identify the differences. *P* values < 0.05 were considered to indicate statistical significance in all tests.

## Results

### The VO_2max_ and exercise intensity

>VO_2max_ was significantly lower in the H trial (41.7 ± 2.2 mL/kg/min) than in the N trial (57.2 ± 2.9 mL/kg/min, *P* < 0.0001). Maximal aerobic power was significantly lower in the H trial (261 ± 28 W) than in the N trial (313 ± 42 W, *P* < 0.0001). Consequently, the absolute workload during the exercise sessions was significantly lower in the H trial (178 ± 19 W) than in the N trial (243 ± 33 W, *P* < 0.0001).

### Cardiorespiratory variables and RPE during exercise

Table 1 shows the cardiorespiratory values during exercise in each trial. The VO_2_, VCO_2_, and SpO_2_ remained significantly lower in the H trial than in the N trial throughout the exercise (main effect for trial, *P* < 0.0001). The H trial showed a significantly higher RER during exercise (main effect for trial, *P* = 0.037). The N trial showed a significantly higher VE during exercise compared with the H trial (main effect for trial, *P* < 0.0001). The HR increased significantly during exercise (sets 2–8 vs. set 1) in both trials (main effect for time, *P* < 0.0001), and the N trial showed a significantly higher HR during exercise (main effect for trial, *P* = 0.001).

**Table 1:** Cardiorespiratory variables during exercise

	N	H	Main effect		Interaction
			Trial	Time	
VO_2_(mL/kg/min)	46.9 ± 2.9	36.6 ± 2.3	*P* < 0.001	n.s.	n.s.
VCO_2_(mL/kg/min)	46.3 ± 3.9	34.1 ± 3.4	*P* < 0.001	*P* < 0.001	n.s.
RER	0.98 ± 0.04	1.02 ± 0.04	*P* = 0.001	*P* < 0.001	n.s.
VE (L/min)	105 ± 17	82 ± 14	*P* < 0.001	*P* < 0.001	n.s.
HR (beats/min)	171 ± 7.2	161 ± 7.7	*P* = 0.001	*P* < 0.001	*P* < 0.001
SpO_2_ (%)	95 ± 1.7	80 ± 3.1	*P* < 0.001	n.s.	n.s.

Values are means ± SE

*VO2* oxygen uptake, *VCO2* carbon dioxide output, *RER* respiratory exchange ratio, *VE* expired minute ventilation, *HR* heart rate, *SpO2* percutaneous oxygen saturation

The average RPE-R during exercise was significantly higher in the N trial than in the H trial (5.0 ± 1.5 vs. 3.6 ± 0.7, respectively, *P* = 0.006). Similarly, the average RPE-L during exercise was significantly higher in the N trial than in the H trial (5.7 ± 1.7 vs. 4.2 ± 1.1, respectively, *P* = 0.0006).

### Blood variables

#### Metabolites

**Fig. 2:**
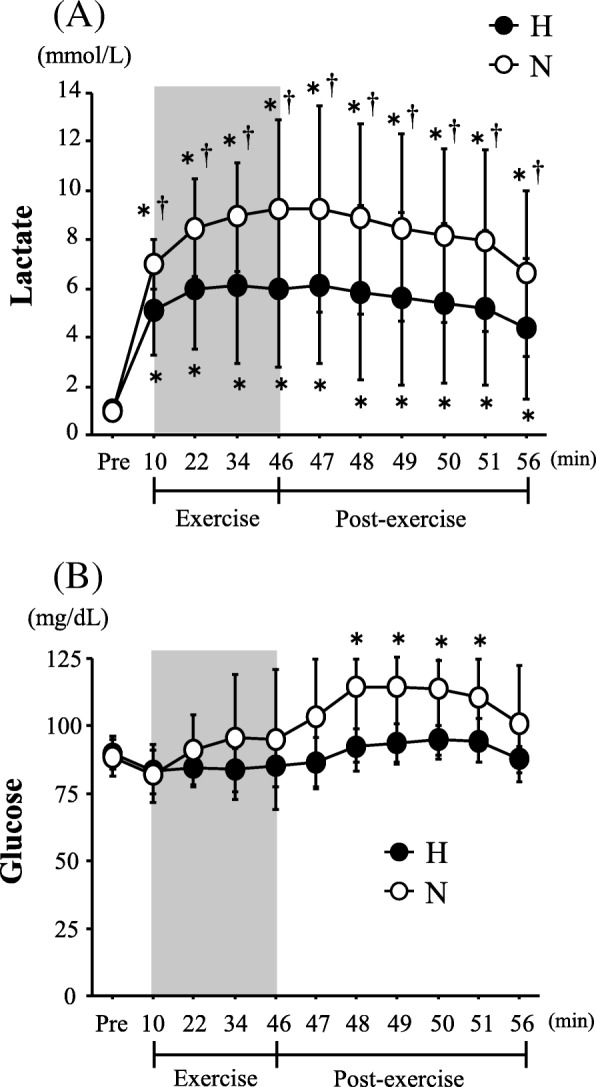
Changes in blood lactate (**a**) and glucose (**b**) concentrations. Values are means ± standard error (SE). The shaded box indicates the duration of exercise. *Significant difference compared with pre-exercise (Pre). ^†^Significant difference between the normoxic (N) and hypoxic (H) trials

Figure 2 shows the changes in the blood lactate and glucose concentrations. The blood lactate concentrations increased significantly with exercise in both trials (main effect for time, *P* < 0.0001). Exercise-induced blood lactate elevations were significantly greater in the N trial than in the H trial (interaction, *P* = 0.002; main effect for trial, *P* = 0.012). The blood glucose concentrations were increased significantly during the post-exercise period in the N trial (main effect for time, *P* = 0.001), and there was a significant interaction between trial and time (*P* = 0.043).

#### Blood pH, HCO_3_^−^, and blood gas kinetics

**Fig. 3:**
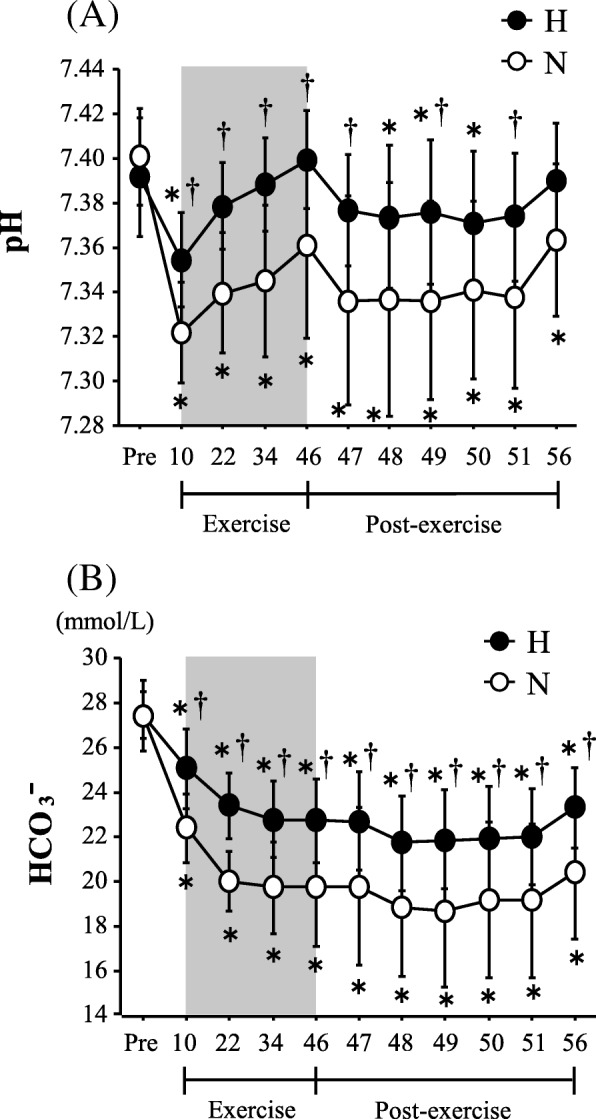
Changes in blood pH level (**a**) and HCO_3_^−^ concentration (**b**). Values are means ± SE. The shaded box indicates the duration of exercise. *Significant difference compared with Pre. ^†^Significant difference between the N and H trials

Figure 3 shows the changes in the blood pH and HCO_3_^−^ level. The blood pH level decreased significantly with exercise in both trials (main effect for time, *P* < 0.0001), and the values were significantly lower in the N trial than in the H trial during exercise and the post-exercise period (interaction, *P* = 0.019; main effect for trial, *P* = 0.019). Both trials showed a significant reduction in the HCO_3_^−^ concentration during exercise and the post-exercise period (main effect for time, *P* = 0.001). The N trial showed a significantly lower HCO_3_^−^ concentration during exercise and the post-exercise period (interaction, *P* = 0.006; main effect for trial, *P* = 0.001).

The pO_2_ decreased significantly during exercise and after exercise in the H trial (main effect for time, *P* = 0.002), whereas no changes were observed in the N trial. Consequently, the pO_2_ remained significantly lower in the H trial during exercise and after exercise (interaction, *P* = 0.018; main effect for trial, *P* < 0.0001). The pCO_2_ decreased significantly during exercise and after exercise in both trials (main effect for time, *P* = 0.002). Exercise-induced reductions in pCO_2_ were significantly greater in the N trial during the post-exercise period (interaction, *P* = 0.861; main effect for trial, *P* = 0.002).

#### Blood K^+^ and Na^+^

**Fig. 4:**
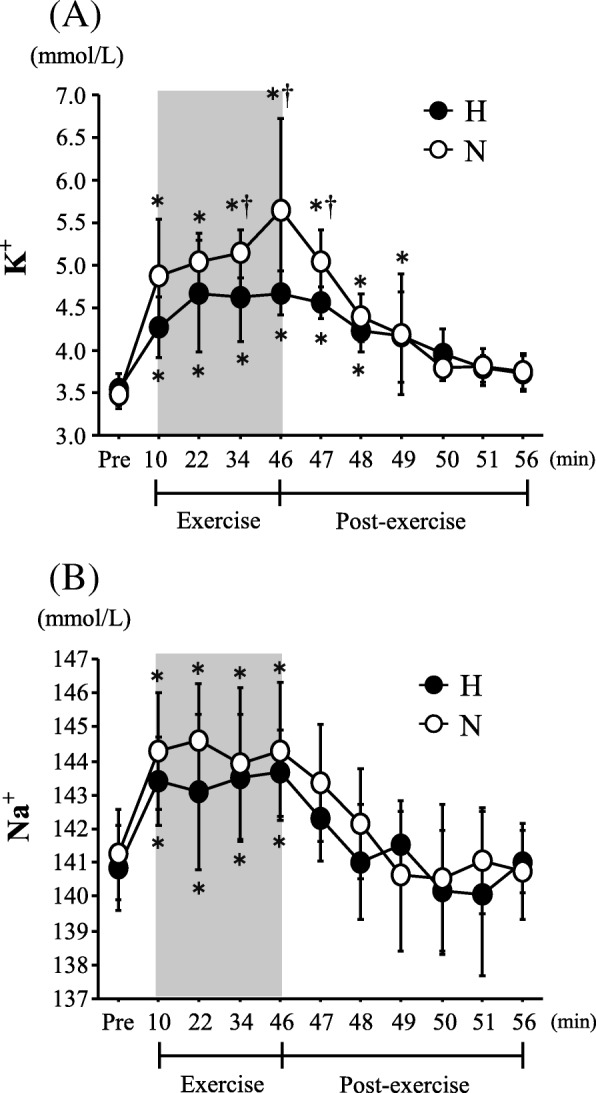
Changes in blood potassium (K^+^) (**a**) and sodium (Na^+^) (**b**) concentrations. Values are means ± SE. The shaded box indicates the duration of exercise. *Significant difference compared with Pre. ^†^Significant difference between the N and H trials

Figure 4 shows the changes in blood K^+^ and Na^+^ concentrations. The blood K^+^ concentration increased significantly during and after exercise in both trials (main effect for time, *P* < 0.0001). In terms of the blood K^+^ concentration, there was a significant interaction (*P* = 0.001), and exercise-induced blood K^+^ elevations were significantly greater in the N trial during exercise (sets 6 and 8) and after exercise (1 min after exercise). The blood Na^+^ concentration increased significantly during exercise in both trials (main effect for time, *P* < 0.0001), whereas there were no significant differences between the H and N trials at any time point (interaction, *P* = 0.506).

## Discussion

Contrary to our hypothesis, exercise-induced elevations in blood lactate and K^+^ concentrations were significantly greater in the N trial than in the H trial, whereas the blood pH level and HCO_3_^−^ concentration were significantly lower in the N trial.

Protons produced in working muscles and released into the bloodstream are buffered mainly by the HCO_3_^−^ buffering system [14, 15]. In the present study, the N trial revealed a greater exercise-induced blood lactate concentration and lower blood pH and level of HCO_3_^−^ concentration during and after exercise. Furthermore, pCO_2_ was significantly lower in the N trial than in the H trial. Accumulation of hydrogen ions decreases pH and subsequently stimulates hyperventilation due to respiratory compensation. Therefore, exercise-induced metabolic acidosis cause lowers pCO_2_ during exercise and the post-exercise period [29], which is consistent with our present findings. We collected venous blood samples to evaluate exercise-induced alterations in acid-base balance, and the same procedure was previously utilized [30, 31]. However, further determination using arterial blood samples or intramuscular information may reveal more details on the regulation of acid-base balance during exercise under hypoxic conditions.

Exercise-induced elevation of blood K^+^ was significantly greater in the N trial throughout the exercise sessions. Exercise-induced metabolic acidosis promotes extracellular accumulation of K^+^, which is controlled by K_ATP_ channels [20–22]. In addition, Street et al. [22] revealed that metabolic alkalosis following sodium citrate ingestion reduced exercise-induced acidosis and extracellular accumulation of K^+^. These findings suggest a close association between the blood pH and the K^+^ concentration. In the present study, the exercise-induced decrease in blood pH and increase in blood K^+^ were augmented in the N trial. Thus, the association between the decreased blood pH and elevated K^+^ observed in the N trial is reasonable. The release of K^+^ is counterbalanced by the activity of the Na^+^/K^+^ pump. However, K^+^ derived from the working muscles during exercise exceeds the capacity of K^+^ re-uptake, and K^+^ generally accumulates in the blood and interstitium [32]. In the present study, the increased blood K^+^ concentration in the N trial has two possible explanations: (1) enhanced release of K^+^ from the working muscle and (2) decreased K^+^ re-uptake during exercise. However, the influence of hypoxia on K^+^ re-uptake has not yet been elucidated. It is therefore plausible that the greater exercise-induced elevation of blood K^+^ in the N trial was influenced by the increased release of K^+^ from the working muscle mediated by lower pH. However, in several previous studies investigating exercise-induced K^+^ kinetics, the samples were collected from interstitial tissue [22, 30]. Because the magnitude of K^+^ kinetics is smaller in the blood than that in the interstitium, caution is necessary in interpreting the results.

Relative exercise intensity during interval exercise (80% of VO_2max_) was matched between the two trials (N trial, 82.0 ± 0.6%; H trial, 80.6 ± 0.7% relative to VO_2max_ under each environment). However, the VE, HR, and blood lactate concentration were significantly lower in the H trial. It has been reported that VE and HR during exercise did not differ significantly between hypoxic and normoxic conditions under the same relative exercise intensity [6, 9], whereas exercise-induced blood lactate elevation was pronounced under hypoxic conditions [10]. These findings were not evident in the present study. VO_2max_ generally decreases under moderately hypoxic conditions [5]. Although the magnitude of the VO_2max_ reduction is especially profound in trained subjects [33, 34], the magnitude in the present study was higher than those documented in previous studies [5, 35, 36]. Therefore, the approximately 30% lower workload in the H trial may have been responsible for the lower acid-base disturbance and concomitant K^+^ elevation, and different outcomes may be found in conditions with greater acid-base disturbance under hypoxic conditions, as reported in previous studies [9, 10].

## Conclusions

High-intensity interval exercise on a cycle ergometer under moderately hypoxic conditions did not elicit the decline of pH and K^+^ elevation compared with an equivalent level of exercise under normoxic conditions in endurance athletes.

## Abbreviations

F_i_O_2_: Fraction of inspired oxygen
H: Hypoxia
HCO_3_: Bicarbonate ion
HR: Heart rate
K^+^: Potassium
N: Normoxia
Na^+^: Sodium
pCO_2_: Carbon dioxide partial pressure
pO_2_: Oxygen partial pressure
RPE: Rate of perceived exertion
VO_2_: Oxygen uptake
VO_2max_: Maximal oxygen uptake
VCO_2_: Carbon dioxide output
RER: Respiratory exchange ratio
VE: Expired minute ventilation
SpO_2_: Percutaneous oxygen saturation
RPE-R: Rating of respiratory strain
RPE-L: Rating of leg strain
